# How to reduce Domestic Violence against married women? a mixed methods study from rural Tamil Nadu

**DOI:** 10.5249/jivr.v14i1.1602

**Published:** 2022-01

**Authors:** Arulmozhi Madhivanan, Amol R Dongre

**Affiliations:** ^*a*^ Department of Community Medicine, Sri Manakula Vinayagar Medical College & Hospital, Puducherry, India.; ^*b*^ Department of Extension Programme (SPARSH), Professor in Community Medicine and Medical Education, Pramukhswami Medical College (PSMC), Karamsad, Gujarat, India.

**Keywords:** Domestic violence, Rural, Spousal abuse, India

## Abstract

**Background:**

Despite government legislations for protection of women, domestic violence (DV) continues to remain as a public health problem in India. Objectives: 1. To find out the prevalence of various types of self-reported DV among married women of 18-45 years of age and to identify its social determinants and their help-seeking behavior. 2. To understand the solutions from key informants’ point of view.

**Methods:**

It was a sequential explanatory mixed methods study design, which consisted of quantitative (Survey) followed by qualitative (Interviews) phase. A representative sample of 360 married women was chosen by two-stage cluster sampling from villages in Tamil Nadu, South India. The female investigator conducted the survey by house to house visit. Post-survey, six key informant interviews were conducted to explore the solutions and suggestions from experts’ point of view. Bivariate and multivariate regression analysis was carried out to identify the significant predictors of DV. Manual content analysis of qualitative data was done.

**Results:**

The overall prevalence of spousal DV was 49.5% [95% CI: 44.3-54.6] in the last one year. In multivariate analysis, two factors namely 'current alcoholism in husband' and 'controlling behavior of husband' were found to be the significant predictors of DV. In order to prevent alcoholism in husband, the key informants suggested deaddiction services and measures to limit access to alcohol. Furthermore, to prevent controlling behavior of husband, the key informants suggested women’s empowerment, employment, helplines, responsible parenting, social change in dowry practice and gender equality.

**Conclusions:**

The prevalence of spousal DV was found to be high. Current alcohol consumption and controlling behavior of the husband were the important determinants of domestic violence. Key informants suggested interprofessional approach consisting of deaddiction services, women empowerment and strengthening of family life to address the problem of DV.

## Introduction

Domestic violence (DV) against women is a neglected, widespread public health problem. Women who are safe at their respective homes often experience violence by their trusted family members, most commonly by husband. Often, it is overlooked as a family problem with its seriousness undermined and unreported. World Health Organization (WHO) reports indicate that at least 35% of women have experienced DV.^1^ Studies across the world show that 10–69% of women report being physically assaulted by an intimate male partner at some point in their lives.^2^ The socio-cultural construct in India allows men to have an upper hand, with women accepting violence and remain as silent sufferers. In spite of government legislations for Protection of Women from Domestic Violence Act, 2005, the prevalence of DV is reported all over India with a national average of 31%, with wide inter-regional differences.^3,4^ Intimate partner violence has serious short- and long-term physical, mental, sexual and reproductive health problems among survivors and their children, leading to high social and economic costs.1 It has been found that women are reluctant to seek help and their quality of life is affected.^5,6^

Although many studies on DV have been conducted in India, only few were from rural south India. Limited is known about the help seeking behavior among the victims of DV. Mere identification of the problems and risk factors alone cannot benefit a society unless it paves a way for solutions. Hence, we conducted this mixed methods study with the objectives of 1. To find out the prevalence of various types of self-reported DV among married women of 18-45 years of age. and to identify its social determinants and their help-seeking behavior. 2. To understand the solutions from key informants’ point of view.

## Methods

**Study setting:** The present study was carried out for a period of one year from January 2018 to December 2018 in the field practice villages of the Rural Health Training Centre (RHTC), attached to the Department of Community Medicine in a tertiary care hospital, Puducherry. The study area consisted of 48 villages covering three primary health centers (Thiruvennainallur, Edaiyar and Sirumadurai) of 92,027 total population in Villupuram district, Tamil Nadu.

**Figure 1:**
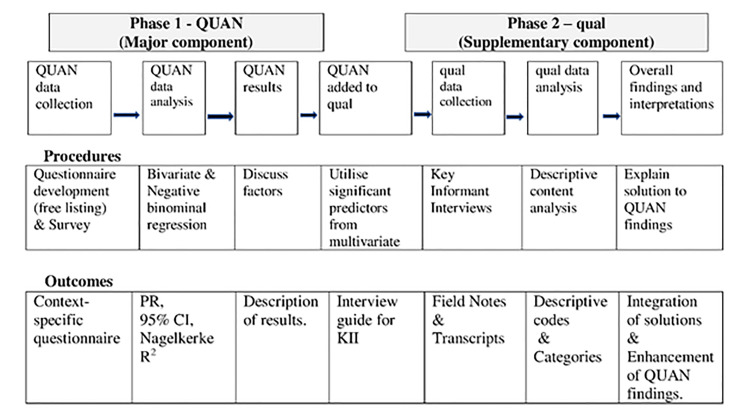
Visual diagram showing sequential mixed method study design.

**Study design:** This was a sequential explanatory mixed methods study design in which quantitative (Survey) phase was followed by qualitative (Interviews) phase.^7^ (QUAN→ qual) (Figure 1).

**Phase-1: Quantitative phase**

**Figure 2:**
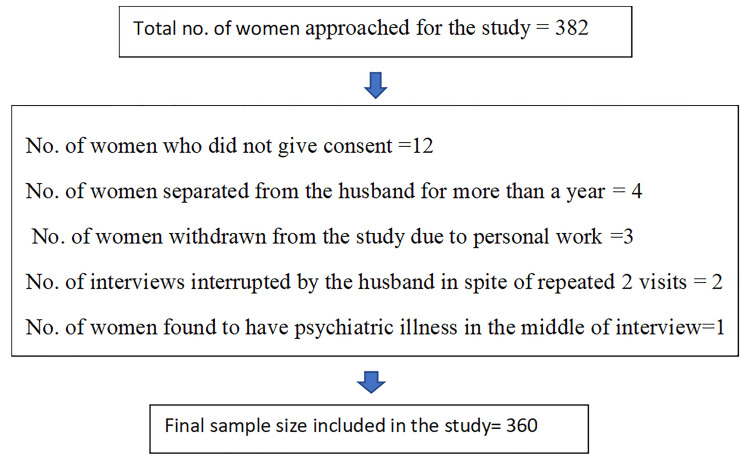
Flowchart of selection of eligible participants.

**Sample size and sampling:** Considering 44.2% prevalence of DV in rural Tamil Nadu from National Family Health Survey (NFHS-4), 7.5% desired precision, design effect-2 and 5% non-response rate, the estimated final sample size was rounded as 360 respondents. Currently married women between the age group of 18 – 45 years from rural field practice area were selected for the study (Figure 2). Two-stage cluster sampling was adopted to select a representative sample of 360 participants. The list of villages and the total population of the study area was obtained from the local block development office. All villages were enlisted in the alphabetical order with their cumulative population. In the first stage, 30 clusters were drawn from the 48 villages using Population Proportional to Size (PPS) method.^8^ In the second stage, 12 women were identified equally from each cluster by 'random walk method' to achieve the desired sample size. In each cluster, a pen was rotated at the center of the village and the investigator proceeded towards the direction (East, West, North, South) of the pen tip, to select the street. The first house in the street of the randomly selected lane was selected by a random method, using the last digit of any currency note taken from the pocket. In this way, consecutively, 12 houses were visited from the first house or till the desired sample size of 12 was reached in each cluster. If there were more than one eligible woman in the household, only one was recruited from each household by a lottery method. ^9^

**Questionnaire development:** The items in the questionnaire were based on variables from literature review and NFHS-4 questionnaire which is a validated structured tool to assess DV. In order to make questionnaire suitable to local context, we did interviews and free list exercise with 15 female health providers (doctors, staff nurses and medical social workers, 5 each).^10^ The most salient items were utilized for options of close ended questions. The questionnaire was pre-tested with local female health service providers for its wording, phraseology and cultural acceptance of questions. The trained female investigator, acquainted as medical officer in RHTC, paid house to house visit and collected data using a pre-designed questionnaire. She was accompanied by female social worker who had experience of working in the field for last ten years. Women were asked questions about experience of spousal violence in last 12 months, ensuring privacy. As per the standard definition, DV includes "violence to women by their spouses and other household members in their lifetime and also in the last 12 months" but in our study we measured only the male spousal violence which is considered as the most common form of DV against women and collected for the last 12 months to minimize recall bias.^4^ In addition to background details, they were asked about types of violence except sexual violence and their help-seeking behaviors. Those women who had a history of DV and asked for help, were referred to respective specialist health services, with a Non-Governmental Organization (NGO) support as back up.

The survey data was entered and analyzed using Statistical Package for the Social Sciences (version 24). Descriptive statistics was calculated for all the background variables in terms of frequency and percentages. In bivariate analysis, we calculated Prevalence Ratio (PR) which estimates the risk of violence. The variables that showed significant association (p value < 0.05) with bivariate analysis and variables of significant importance from previous literature were selected for multivariate analysis. Multivariate analysis using negative binomial regression was carried out. The outcome variable is a categorical dichotomous variable- "presence or absence of any one form of violence" which was recorded as count of presence or absence of any one type of domestic violence. Since the overall prevalence of any type of domestic violence was high (50%), negative binomial regression (Cox regression) was carried out in multivariate analysis by adding 'Zero' as the time component. All the assumptions were satisfied and the final model was developed by forward selection process. We included 17 independent variables such as wife’s age, education, occupation, socioeconomic status, caste, husband’s education, debt/loan, alcohol and smoking habit in husband, age at marriage, duration of marriage, arranged marriage, dowry, type of family, number of children, living house and controlling behavior of husband for bivariate analysis. "Controlling behavior" included six items based on NFHS-4, such as limiting access to family and friends, continued watchfulness and lack of trust including on financial issues.^4^ Multicollinearity and interaction among the independent variables were ascertained and tolerance level was checked. Further using 'R' software, three outliers were identified in our data analysis that skewed the data. The outlier form numbers were identified, the forms were traced and checked for typographical errors. As the information were entered correctly, the three-outlier data were removed from the analysis for negative binomial regression. The coefficient of determination (R^2^) was used to determine the goodness-of-fit statistic for the model and the statistical significance level was set at 5 percent. 

**Phase – II: Qualitative phase: One-to-one Interviews**

After survey data analysis, key informant interviews were done with female key informants (Gynaecologists, staff nurses and medical social workers, two each), from the tertiary hospital who were vocal, willing, and assumed to be knowledgeable of topic. A convenient type of purposive sampling of six Key Informant Interview was chosen, as we achieved saturation of information and no new information being added by the sixth interview.^11^ An interview guide was prepared with a broad open-ended question utilizing the quantitative findings and piloted in two female doctors, to explore the solutions from health worker’s point of view. These questions were focused on two significant determinants namely "alcoholism and controlling behavior of the husband" found in multivariate analysis. Topic was informed well in advance to the key informants and all agreed to participate in the interview as they have frequently seen, women with DV in their career. After obtaining informed consent, the trained investigator conducted interview at the time and place convenient to the participants. Each interview lasted for about 20-30 minutes. The interview was audio-recorded and field notes were taken simultaneously during the interview. Sufficient prompts were utilized to get the in-depth information, for additional clarification and to avoid being deviated from the topic. Debriefing was done at the end of each interview for participant validation and later transcribed. The final report was rechecked and given back to the participants to confirm for its accuracy. Manual descriptive content analysis was performed to generate the key themes.^12^ Similar codes were merged to form the categories and themes. Statements in italics represents the direct quotes from the participants. First author carried out the content analysis and second author verified it to bring interpretative credibility. 

The Good Reporting of A Mixed Methods Study (GRAMMS) guidelines was used for reporting the mixed methods study findings.^13^

## Results

Out of 360 women, 24 respondents who were found to be anaemic and self-reporting with other illnesses were referred to medical treatment and 13 alcoholic husbands were referred for deaddiction services. For the three women, who had history of DV, the female social worker number was provided to contact in case of need of any assistance.

The median age of the respondents was 32 (IQR 27-38) years. About 167 (46.4%) women were in the age group of 26-35 years and majority (318) (88.3%) were able to read and write. Their median year of education was 10 (IQR 7-12) years. More than half (197) (54.7%) women were home makers. Other background details are given in Table 1.

**Table 1:** Bivariate and multivariate analysis of determinants of domestic violence (N=360).

Variables	N (%)	H/O Domestic violence n (%)	Unadjusted PR(95% CI)	Adjusted PR(95% CI)
**Age of wife**				
18-25	71 (19.7)	38 (53.5)	1.15 (0.64-2.07)	0.99 (0.95-1.02) ≠
26-35	167 (46.4)	79 (47.3)	0.90 (0.56-1.43)	
36-45	122 (33.9)	61 (50.0)	1	
**Educational status of wife**				
Illiterate	42 (11.7)	28 (66.7)	2.24 (1.14-4.41)*	1.16 (0.75-1.81)
Literate	318 (88.3)	150 (47.2)	1	1
**Socioeconomic status (Modified BG Prasad classification)**				
Class 4,5	198 (55.0)	101 (51.0)	1.15 (0.76-1.74)	0.92 (0.66-1.29)
Class 1,2,3	162 (45.0)	77 (47.5)	1	1
**Educational status of husband**				
Illiterate	43 (11.9)	24 (55.8)	1.34 (0.70-2.54)	1.10 (0.69-1.75)
Literate	317 (88.1)	154 (48.6)	1	1
**Alcohol use in husband**				
Current user	175 (48.6)	120 (68.6)	7.32 (4.32-12.43)*	2.18 (1.38-3.44)*
Past user	63 (17.5)	30 (47.6)	3.05 (1.59-5.85)*	1.66 (0.96-2.86)
Nonuser	122 (33.9)	28 (23.0)	1	1
**Smoking habit in husband**				
Current user	128 (35.5)	90 (70.3)	3.82 (2.40-6.10)*	1.30 (0.92-1.83)
Ever user	15 (4.2)	5 (33.3)	0.81 (0.27-2.44)	0.83 (0.33-2.10)
Nonuser	217 (60.3)	83 (38.2)	1	1
**Duration of marriage (years)**				
<1	10 (2.8)	2 (20.0)	1	1
1-20	288 (80.0)	149 (51.7)	4.29 (0.90-20.54)*	2.05 (0.45-9.42)
21-40	62 (17.2)	27 (43.5)	3.09 (0.61-15.73)	1.95 (0.38-10.07)
**Arranged marriage**				
Yes	283 (78.6)	132 (46.6)	1	1
No	77 (21.4)	46 (59.7)	0.57 (0.33-1.00)*	1.04 (0.71-1.51)
**Dowry asked after marriage**				
Yes	54 (15.0)	36 (66.7)	2.31 (1.26-4.25)*	1.06 (0.71-1.58)
No	306 (85.0)	142 (46.4)	1	1
**Type of family**				
Nuclear	187 (51.9)	99 (52.9)	1.18 (0.59-2.36)	1.03 (0.62-1.72)
Joint	134 (37.2)	60 (44.8)	1.91 (0.96-3.80)*	0.94 (0.55-1.60)
Three generation	39 (10.8)	19 (48.7)	1	1
**Living house**				
Own	340 (94.4)	163 (47.9)	1	1
Rent	20 (5.6)	15 (75.0)	3.26 (1.16-9.16)*	1.59 (0.92-2.75)
**Controlling behavior of husband**				
0 (good)	202 (56.1)	65 (32.2)	1	1
1-2 (poor)	113 (31.4)	73 (64.6)	3.85 (2.37-6.25)*	1.76 (1.25-2.49)*
3-6 (worse)	45 (12.5)	40 (88.9)	16.86 (6.36-44.72)*	2.06 (1.33-3.19)*

Note: ≠Continuous variable *p value < 0.05

Overall, among the 360 respondents, 178 (49.4%, 95% CI: 44.3-54.6) women experienced at least one type of violence and 82 (22.8%) women suffered both physical and emotional violence in the past one year before the survey. About 131 (36.4%) women experienced only emotional violence and 129 (35.8%) experienced physical violence alone. 

Among the victims of physical violence, 101 (28.1%) women experienced slapping, 52 (14.4%) reported pushing, shaking, or throwing something over them. Furthermore, 30 (8.3 %) women reported severe violence such as husband attempting to choke or burn, threaten, or attack with a knife or other weapon. The most common type of injury reported was cut, bruises or aches by 36 (10%) women followed by deep wounds, broken bones, broken teeth, or other serious injury by eight (2.2%) women. In addition, seven (1.9%) women also reported grievous injuries like eye injury or dislocation. Among women who experienced emotional violence, 96 (26.7%) women reported that their husband had humiliate her in front of others, 92 (25.6%) women reported that their husband insulted or made them feel bad about themselves and 23 (6.4%) women mentioned that their husband threatened to harm them or their beloved ones.

Among the 178 victims, 86 (48%) women reported that they informed someone about the violence and 85 (47.8%) did seek help to curtail the violence. Of various sources of help, 69 (38.8%) approached their own family, 7 (3.9%) complained to police and 1 (0.6%) sought help from doctor. 

In final model of multivariate analysis, after adjusting for all confounders in the negative binomial regression, the two factors namely 'current alcoholic husband' and 'the controlling behavior of husband' were found to be significant predictors of DV. Among the married women who responded the prevalence ratio of DV was 2.18 times (95% CI: 1.38-3.44) higher in women with current alcoholic husband compared to those with husbands who never consumed alcohol. Women living with a husband who showed 3-6 items of controlling behavior was 2.06 times (95% CI:1.33-3.19) at higher risk of experiencing domestic violence compared to husbands who did not have any controlling behavior. This model was fitting well, evident from Hosmer-Lemeshow Test as it showed insignificant result. The 42.4% prediction of DV has been explained by the Nagelkerke R^2^ model (Table 1). The solutions suggested by the key informants for prevention of alcoholism and controlling behavior of husband are given in Table 2.

**Table 2:** Suggested solutions by key informants for prevention of alcoholism and controlling behavior in husband (N=6).

Themes	Categories	Suggested measures
**Alcoholism**	**Deaddiction services**	1. Screening OPD for alcoholism.
		2. Increase deaddiction centres and offer psychiatric support.
		3. Motivation
	**Limit access to alcohol**	1. Restrict liquor sale timings & limit alcohol buying capacity per person.
		2. Enforce alcohol ban.
		3. Liquor shops should be away from residential areas.
		**"Government should not consider alcohol as a source of revenue; instead if there is political commitment definitely, they can ban alcohol"**
	**Social change through awareness**	1. Restrict alcohol peer pressure in hotels/meetings/celebrations
		2. Restrict alcohol drinking scenes in films and social media.
		3. Awareness through social media, school curriculum and NGO initiatives
**Controlling behavior of husband**	**Education and Occupation for women**	1. Educate and empower women through education to fight for their rights.
		2. Employment opportunities for rural women to make financially independent.
		**"If women are educated and go to work, they can be confident and see other women and able to fight for their rights"**
	**Strengthen family life and support**	1. At home, family should teach male child to respect females.
		2. Teach female child to adjust in new family, maintaining their rights and respect.
		3. Parents should not consume alcohol in home
		4. Pre-marital counselling of couple on their desired roles in family building.
	**Helplines for victims**	1. Awareness about helplines/ counselling/referral centres
		2. Develop women aid organizations and support groups.
		3. Strengthen one stop centre for victims in health facility.

## Discussion

We found that half of the participating women have experienced at least one form of DV from the husband in last one year. Nearly half of the victims (48%) attempted to seek help from informal sources. In multivariate analysis, factors such as 'current alcoholism in husband' and 'controlling behavior of husband' were found to be significantly associated with the experience of DV in women. Key informants suggested measures such as increase in deaddiction services, limiting the accessibility and availability of alcohol, social change through awareness. They also felt that controlling behavior of husband could be addressed through women empowerment, and family life strengthening. 

The present study has shown that the overall prevalence of self-reported spousal DV in the last one year in study area was 49.4 percent which was close to the figure (44%) reported in NFHS-4 for rural Tamil Nadu. Previous studies conducted in rural Tamil Nadu and neighboring rural Puducherry have reported higher figures ranging from 57% to 77percent.^14,15,16^ This difference in prevalence might be due to the sampling variation in the surveys. States like Kerala and Tripura with high female literacy rate, show lesser prevalence of DV. Notably, Tamil Nadu despite being a state with high female literacy (72.9%) still reported higher prevalence of DV,^4^ reflecting the importance of overall status of women in society. 

We found that among the women who experienced DV, nearly half sought help to stop the violence. Chauhan et al in rural Hyderabad observed the same behavior.^17^ The findings of the present study and past studies suggests that women with husband having greater controlling behavior and alcoholic addiction are more likely to seek help.^18^ we also found that majority preferred informal sources of help similar to NFHS-4 findings.^4^ Women are less likely to approach formal institutions unless DV is of long standing or of increased severity, resulting in injuries or endangering their lives.^19^ Henceforth, as recommended in key informant interview support from parents, increasing awareness of helplines and accessibility to women aid organizations, and strengthening one stop crisis centre in all hospitals can result in improved help seeking behavior. 

Furthermore, rural areas showed a higher trend of prevalence of domestic violence than urban areas.^4^ Noteworthy, in rural areas the immediate helping hand in the community would be the neighboring people. Empowering the society against DV by community organizing efforts and enhanced social participation can reduce such violence in the community.^20^ We found association between current alcohol use in husband DV. A similar association was consistently observed in past studies in India.^14,16,21-25^ It has been observed that increase in frequency of alcohol intake considerably increase the prevalence and severity of violence.^4,26^ Key informants in the present study strongly recommended government to ban or limit the sale of alcohol to reduce the problem of social evil of domestic violence. Community action has been found to influence alcohol policy and reduced liquor availability resulting in reduced DV.^27^

In addition, we found association between controlling behavior of the husband and DV, which is consistent with past studies in Calcutta, and Madhya Pradesh.^26,28^ In patriarchal society, husband assumes to have the right to control or beat wife as a way of disciplinary action and women justify wife beating, provides a favorable environment for DV.^29^ DV is relatively low in matriarchal societies like Kerala and Khasi community in Meghalaya.^4,30^ Key informants suggested that empowering women, and strengthening family life would possibly reduce DV.

As the present study was a sequential mixed method study, it offered the figures of DV, determinants and offered the solutions to problems in the given context. The study was based on a representation sample and qualitative data was collected, using the context-specific questionnaire and qualitative information was collected till the point of saturation. The major limitation of the study is that we have included only spousal violence and sexual abuse was not measured as people may not find comfortable to disclose very sensitive issues. However, there might be under-reporting or participants might have reported the less severe form of violence due to social desirability or fear of reporting violence to an investigator. Being a cross sectional study design, temporality of association between risk factors and domestic violence could not be ascertained.

## Conclusion

The prevalence of spousal DV in the past one year was high in this rural study setting. Emotional violence was relatively more prevalent as that of physical violence which necessitates focus on mental health. Current alcohol consumption and controlling behaviour of the husband were the important determinants of domestic violence. Interprofessional approach consisting of deaddiction services, women empowerment and strengthening of family life has been suggested by the key informants to address the problem in the context. Henceforth, the recommendations are all female patients should be routinely screened for DV and male patients for alcohol abuse in primary health care settings. For this the health professionals and community key informants should be adequately trained in identification of victims and counselling skills. Community-based mental health programs should be developed and strengthened. Women’s support groups and networks should be developed at village level. Behavior change communication for gender equality and substance abuse, should be encouraged and instilled in the young minds of school going children. Thus, overall, it would require a multipronged strategy for the development of social support and overall change in societal mindset. 

**Acknowledgement**

We acknowledge "Nehru Yuva Kendra" NGO, Villupuram who agreed to be a helping hand in our study in need of women support. We thank all the key informants for their valuable inputs.
